# Supplementary material for: Rapid in situ carbon-13 hyperpolarization and imaging of acetate and pyruvate esters without external polarizer
Source: Commun Chem. 2024 Oct 23;7:240. doi: 10.1038/s42004-024-01316-x (PMC11499913; doi:10.1038/s42004-024-01316-x)
Supplement: Supplementary file 2 — Supplementary information [file 42004_2024_1316_MOESM2_ESM.pdf]

Supplementary Information

Rapid In Situ Carbon-13 Hyperpolarization and Imaging of Acetate and Pyruvate Esters without External Polarizer

Obaid Mohiuddin,[1] Henri de Maissin,[1,2] Andrey N. Pravdivtsev,[3] Arne Brahms,[4] Marvin Herzog,[1,2] Leif Schröder,[5] Eduard Y. Chekmenev,[6] Rainer Herges,[4] Jan-Bernd Hövener,[3] Maxim Zaitsev[1], Dominik v. Elverfeldt,[1] Andreas B. Schmidt.[1,2,6,*]

[1] Division of Medical Physics, Department of Diagnostic and Interventional Radiology, University Medical Center Freiburg, Faculty of Medicine, University of Freiburg, Killianstr. 5a, 79106 Freiburg, Germany.

[2] German Cancer Consortium (DKTK), partner site Freiburg, German Cancer Research Center (DKFZ), Im Neuenheimer Feld 280, Heidelberg 69120, Germany.

[3] Section Biomedical Imaging, Molecular Imaging North Competence Center (MOINCC), Department of Radiology and Neuroradiology, University Medical Center Schleswig-Holstein and Kiel University, Am Botanischen Garten 14, 24118 Kiel, Germany.

[4] Otto Diels Institute for Organic Chemistry, Kiel University, Otto-Hahn-Platz 5, 24118, Kiel, Germany.

[5] Division of Translational Molecular Imaging, German Cancer Research Center (DKFZ), Im Neuenheimer Feld 280, 69120 Heidelberg, Germany

[6] Integrative Biosciences (Ibio), Department of Chemistry, Karmanos Cancer Institute (KCI), Wayne State University, 5101 Cass Ave, Detroit, MI 48202, United States.

* andreas.schmidt@uniklinik-freiburg.de

Table of Contents

1. Reactor 3

Reactor Design: 3

Pressure simulation of the reactor: 4

2. Parahydrogen enrichment fraction 4

3. ^{13}C Hyperpolarization of 20 mM ethyl acetate as a function of catalyst concentration 5

4. ^{13}C Polarization as a function of number of refocusing pulses per evolution time interval of the SOT sequence 6

5. ^{13}C Polarization as a function of precursor concentration 7

6. T_1 measurement of ethyl [1-^{13}C] pyruvate-d_6 7

7. Polarization transfer efficiency 8

8. ^{13}C Hyperpolarization quantification using external ^{13}C reference solution 9

9. References 10

1. Reactor

Reactor Design:

A reactor was custom-designed for this study (Autodesk Inventor Professional, Autodesk, USA). It was made from Polyether Ether Ketone (PEEK) via Computer numerical control (CNC) technology (mechanical workshop, University Medical Center Freiburg, Germany). All sealings were from perfluoroelastomeric compounds (FFKM). Supplementary Figure 1a illustrates the reactor, equipped with an attachment for positioning it at the isocenter of the MRI setup and the ^{13}C-^{1}H volume coil (V-XLS-HL-070-01349 V01, Rapid Biomedical GmbH, Germany). The coil, featuring an inner diameter of 71 mm, seamlessly accommodates the 70 mm outer diameter of the reactor, ensuring that the reaction chamber was centered at the symmetry axis of the coil. The reactor's internal temperature can reach up to 75°C, with continuous water circulation (PC300 immersion circulator, ThermoFisher Scientific, USA) set to 90°C. This temperature difference results from the thickness of the material wall between the water and reaction chamber (approximately 11 mm).

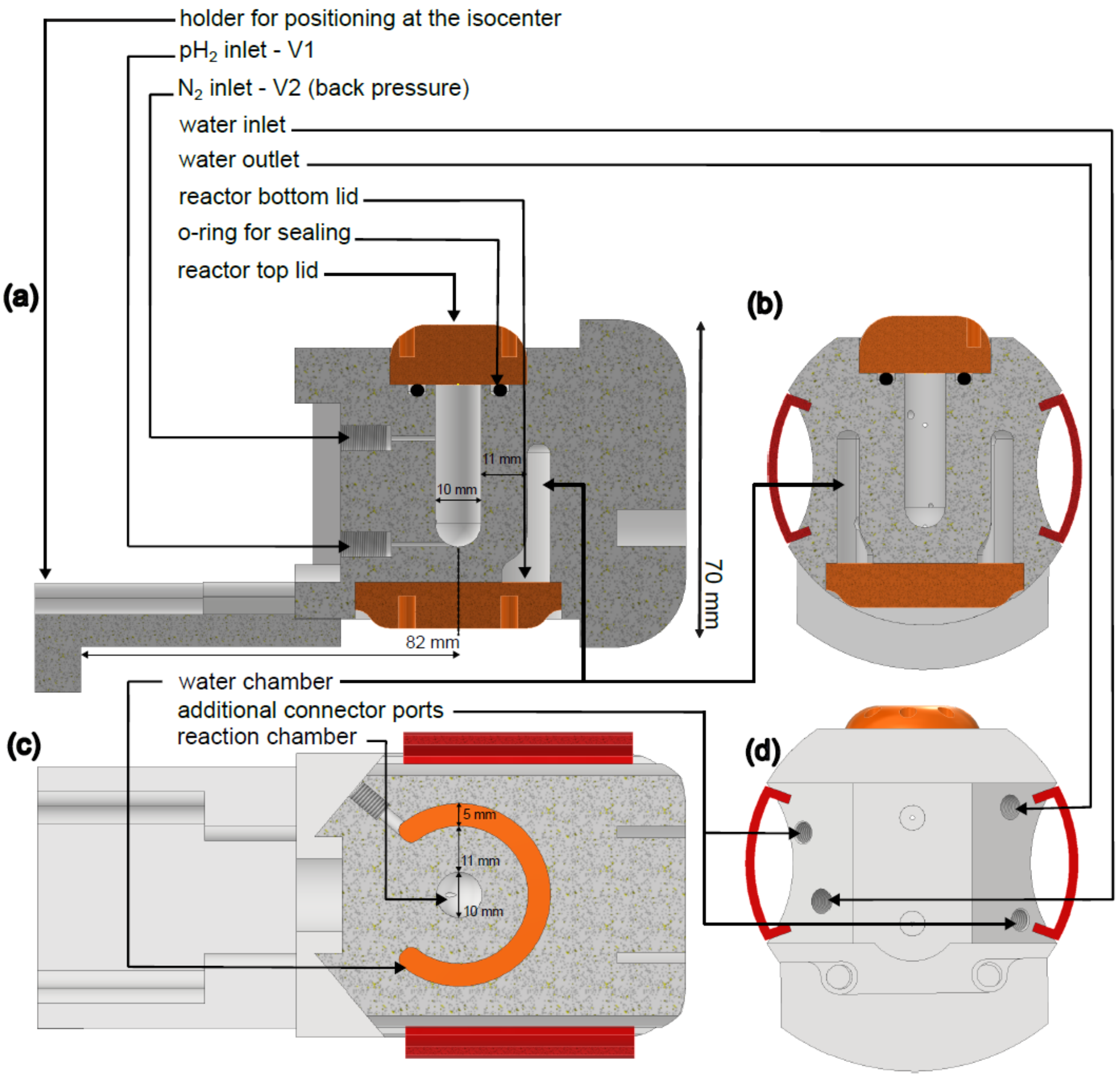

Supplementary Figure 1: CAD drawing of the reactor used for the hyperpolarization experiments. (a) Cross-section of the side view of the reactor showing the size of the reaction chamber which hosted 1-mL catalyst-substrate solution during hydrogenation and spin order transfer (SOT). (b) Back view cross-section to visualize how the water chamber encapsulates the inner chamber to warm the

precursor solution. (c) Top cross-section view showing the relative size of the inner reaction and outer water chamber. (d) Front view of the reactor where all the connecting ports are located, the middle two inlets are for introducing pH_2 and N_2 (also shown in Supplementary Figure 1a); the ones on the sides are for filling and extracting solution and for flowing heated water through the heating chamber. The two curved pieces on the sides (indicated in red) are for guiding the tubes connected to the ports. V1 and V2 refer to the magnetic valves which controlled the pH_2 and N_2 gas flow (see Figure 1 in main text).

Pressure simulation of the reactor:

A comprehensive pressure simulation was conducted for the reactor using finite element method analysis. In the Autodesk inventor professional, we applied an initial pressure of 50 bar to all inner walls, including the inlet and outlet boreholes and cap of the reactor touching the pressurized H2 gas during the experiments, and are considered to be closed, following the experimental conditions. The simulation strain and stress affirm the reactor's structural integrity, demonstrating its capacity to withstand pressures exceeding 50 bar. Based on the tensile strength of the PEEK material of the reactor, the simulation suggests no deformity till 162 bar (≈16.24 MPa), shown in the deformity scale on the left.

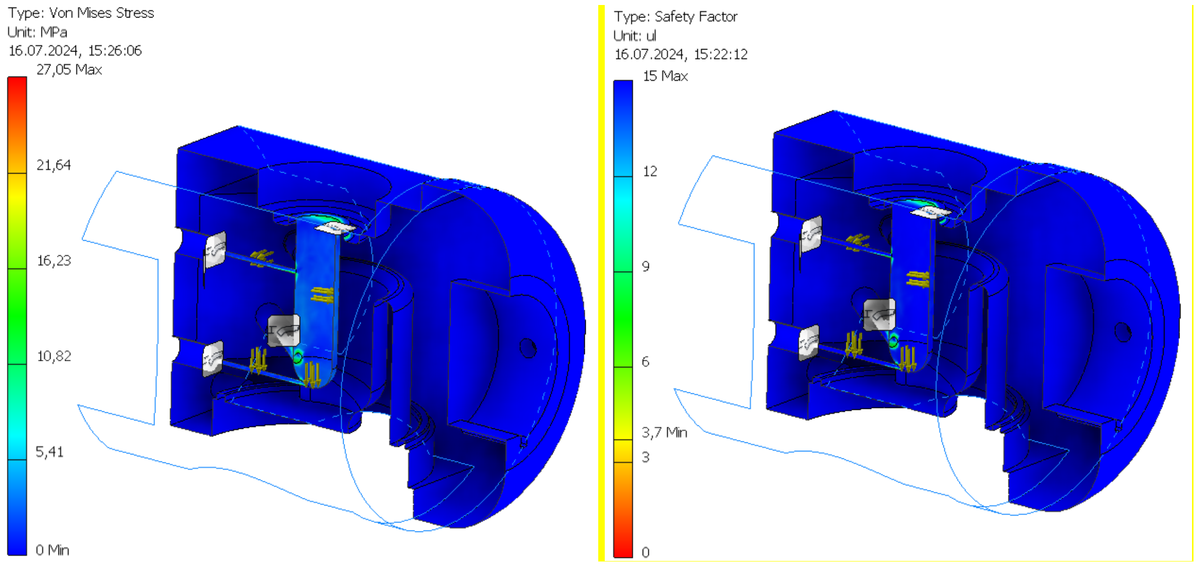

Supplementary Figure 2: Pressure simulation of the reactor at 50 bar left and safety factor simulation on the right, using Autodesk Inventor Professional 2024. Here, a safety factor 1 would indicate that the material fails at that point.

2. Parahydrogen enrichment fraction

To quantify the parahydrogen enrichment, a single NMR tube underwent successive pressurization to 5 bar with N_2, room temperature hydrogen (rtH_2), and parahydrogen (pH_2). Measurements were conducted using a benchtop spectrometer at 1T (Magritek SpinSolve carbon 60). Gas signals were acquired using 90° flip-angle RF pulses, 128 averages, and a repetition time of 300 ms to eliminate

background signals stemming from the glass and impurities, the free-induction-decay (FID) signal produced by N_2 was subtracted from both rtH_2 and pH_2 FID signals before Fourier transformation. The quantification of parahydrogen enrichment was determined using the following equation:

$$f = 100 - 75 \cdot \frac{S_{\mathrm{pH2}}}{S_{\mathrm{rtH2}}} \qquad (1)$$

Where, S_{pH2} denotes the area under the curve for pH_2. S_N is for air, which in our case is nitrogen gas, and S_{rtH2} is for the hydrogen gas stored at room temperature.

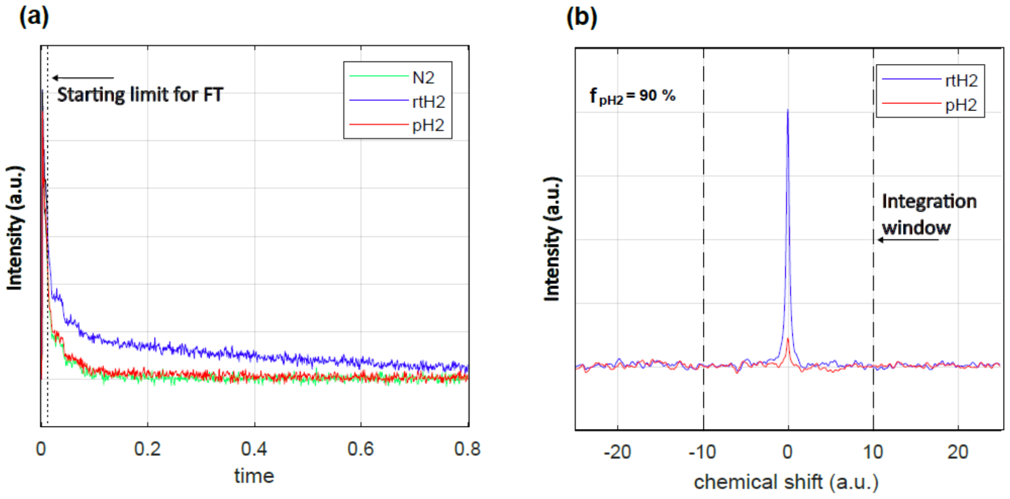

Supplementary Figure 3: Evaluation of the parahydrogen enrichment. (a) Time domain NMR signal produced by N_2, thermally-equilibrated room temperature hydrogen (rtH_2) and enriched parahydrogen (pH_2). The signal acquired from N2 was subtracted from signals recorded from the rtH_2 and pH_2 samples to remove background. The first data before the indicated black dashed vertical line were discarded before the Fourier transformation (FT) to reduce baseline distortion caused by acoustic ringing phenomenon[1]. (b) The processed rtH_2 and pH_2 frequency domain signal. The determined enrichment fraction was ≈90±5%.

3. ^{13}C Hyperpolarization of 20 mM ethyl acetate as a function of catalyst concentration

Investigating the effects of concentration of the rhodium-based catalyst on ^{13}C polarization levels, solutions containing VA were prepared with catalyst concentrations of 1, 2, 5, 10, and 20 mM. The data suggests that the catalyst concentration did not significantly impact the polarization yield in the investigated range (see Supplementary Figure 4).

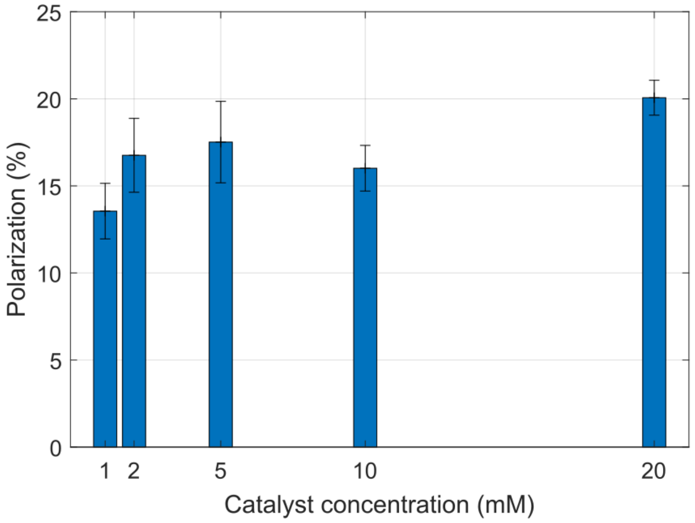

Supplementary Figure 4: 1-^{13}C HP EA as a function of concentration of catalyst. The concentration of EA precursor was 20 mM for all experiments.

4. ^{13}C Polarization as a function of number of refocusing pulses per evolution time interval of the SOT sequence

It has been shown that interleaving multiple refocusing pulses during the spin-order-transfer (SOT) sequence can reduce the loss of coherence during the evolution intervals[2]. In order to determine the optimum conditions for our setup and SOT, the ESOTHERIC[3] sequence was implemented with one to five simultaneous ^{1}H and ^{13}C composite refocusing pulses per each evolution time interval. All other parameters were set constant as described in the main text. It is concluded that 2-refocusing pulses were optimal, reaching the highest 1-^{13}C polarization levels for EA using the ESOTHERIC sequence[4]. Consequently, all the experiments reported in the main text were performed using two 1H/13C composite refocusing pulses per time interval (as displayed in main text Figure 2).

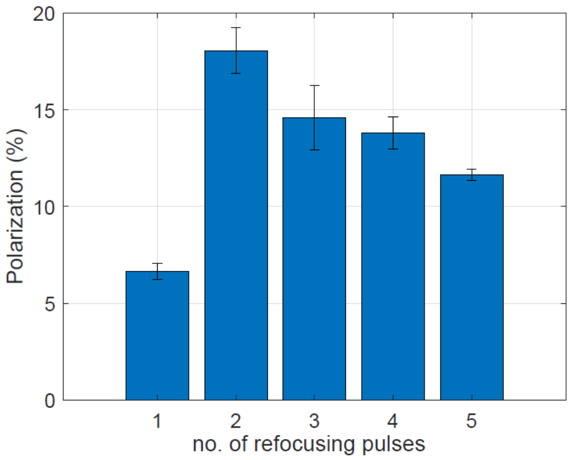

Supplementary Figure 5: 1-^{13}C Polarization of EA as a function of the number of refocusing pulses per time interval of the ESOTHERIC sequence. The error bars shown are three different experiments.

5. ^{13}C Polarization as a function of precursor concentration

The SAMBADENA experiments were conducted with different concentrations of vinyl-acetate-d_6 while maintaining a precursor-to-catalyst ratio of 16 and using otherwise identical experimental conditions. We found that evaluated 1-^{13}C polarization of ethyl acetate-d_6 appeared to decrease with precursor concentration (see Supplementary Figure 6). However, the apparent decrease of polarization with precursor concentration is likely attributable to incomplete hydrogenation, as our quantification had considered complete turnover (main text equation 2).

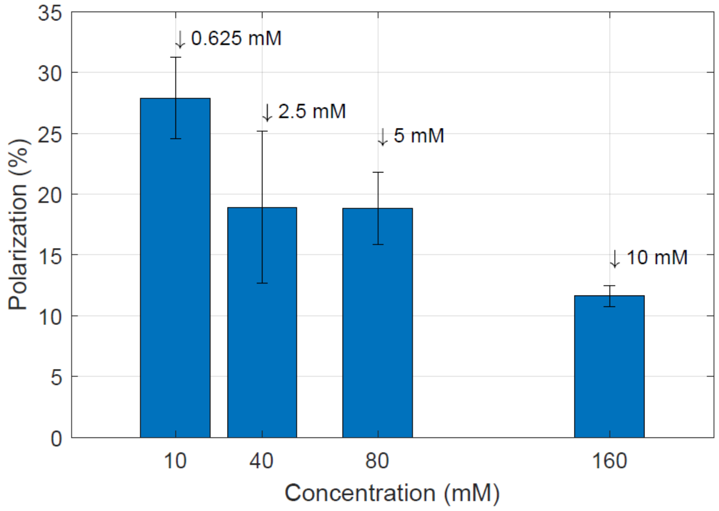

Supplementary Figure 6: 1-^{13}C polarization of ethyl acetate-d_6 as a function of concentration of precursor. The values on top of the bars (indicated with arrows) show the used concentration of the catalyst.

6. T_1 measurement of ethyl [1-^{13}C] pyruvate-d_6

Longitudinal relaxation of ^{13}C hyperpolarized ethyl [1-^{13}C] pyruvate-d_6 was investigated (Supplementary Figure 7a). Interestingly, we observed a spontaneous side-arm cleavage yielding pyruvate, similar to a previous study using EP[5]. Note that in the former study, this effect was attributed to the presence of trace amounts of water. Consequently, to accurately determine the T_1 relaxation times, we developed a kinetic model. This model utilized two linear coupled ordinary differential equations to fit the evolution of the hyperpolarized signals. :

$$\begin{aligned}\frac{d[EP]}{dt} &= -\left(K_{EP-P} + \frac{1}{T_{EP}}\right) \cdot [EP] \\ \frac{d[P]}{dt} &= K_{EP-P} \cdot [EP] - \frac{1}{T_P} \cdot [P]\end{aligned} \quad (2)$$

K_{EP-P} is the rate constant for the cleavage reaction from EP to pyruvate, found to be $K_{EP-P} = 0.044$ by fitting the model. The longitudinal relaxation time constants for EP and pyruvate, T_{EP} and T_P, were found to be 22.4 ± 5.28 s and 23 ± 1.7 s, respectively. The relatively short T_1 values were potentially caused by impurities introduced during the preparation of the reaction solution, potentially due to N_2 bubbling used for degassing. Note that Glöggler and colleagues have presented a 69 s T_1 of EP at the same magnetic field under similar conditions.[2]

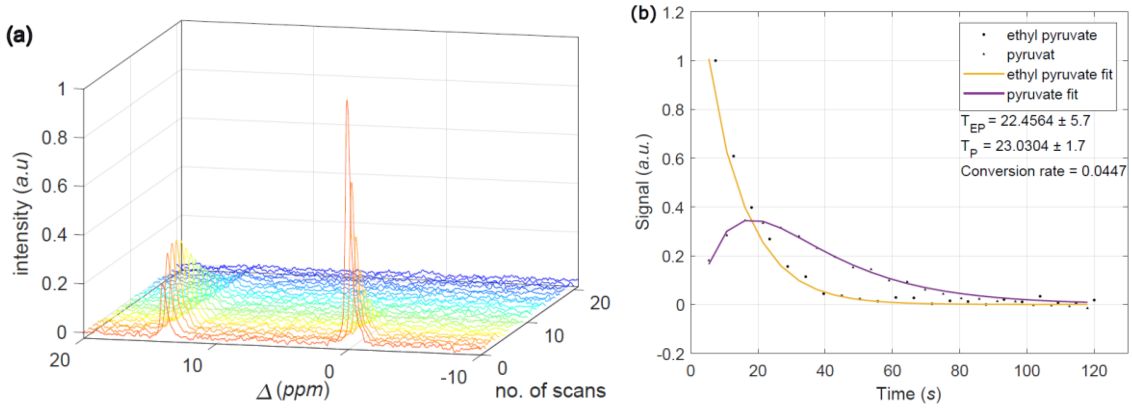

Supplementary Figure 7: Monitoring longitudinal magnetization of 80 mM ethyl-[1-^{13}C]-pyruvate-*d*$_6$ (EP) after hyperpolarization in the preclinical 7T MRI system. Note that experimental parameters were the same as described in the main text. (a) Waterfall plot of the ^{13}C T_1 measurement acquired with 12s repetition time and a 9° flip angle. (b) Integrated ^{13}C signals (dots) and fitted curves (lines). The extracted T_1 relaxation times for ethyl pyruvate and pyruvate were T_{EP} = 22.4 ± 5.28 s and T_P = 23 ± 1.7 s, respectively.

7. Polarization transfer efficiency

The polarization transfer efficiency was assessed using 80mM VA and 5mM catalyst in 1 ml acetone respectively, at T=90°C and p=25 bar pH_2 pressure after 7 s total hydrogenation time (*i.e.*, 5 s bubbling plus a 2 s delay). To quantify 1H polarization, the out-of-phase echo (OPE) sequence was used (Supplementary Figure 8a). Note that direct acquisition of PASADENA signals was not possible as the anti-phased signals collapse and cancel each other out due to the poor field homogeneity of the MRI system. Hence, the choice of the OPE sequence was motivated by the aim to capture signals for both protons (H_a and H_b) of EA. To quantify ^{1}H polarization, the H_a signal was integrated and subsequently multiplied by two, considering the theoretical limitation in transfer efficiency of the OPE, which is 50%. Additionally, the signal of a 1-ml sample of protonated water was acquired as a reference, utilizing a Point RESolved Spectroscopy (PRESS) sequence with same acquisition parameters. The calculated ^{1}H polarization was approximately ≈30%.

For the determination of ^{13}C polarization, 1 ml of 80 mM VA was employed under identical conditions (Supplementary Figure 8b). A single scan of acetic acid-1-^{13}C (CAS: 4563-79-7, Sigma-Aldrich, USA) served as the reference. The calculated ^{13}C polarization of EA was approximately ≈20%. Hence, we estimated a polarization transfer efficiency of about ≈66%.

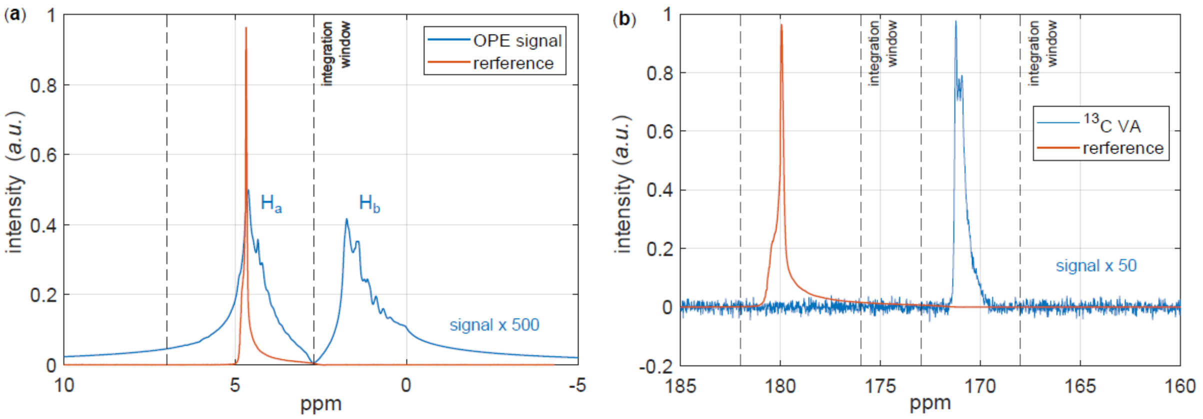

Supplementary Figure 8: Polarization transfer efficiency evaluated using 80mM vinyl acetate-d$_6$ and a 7-seconds hydrogenation time. (a) Blue curve shows the OPE signal of EA and orange curve shows the reference spectrum of protonated water. (b) Blue curve shows the ^{13}C signal of 80mM EA and orange curve shows the reference spectrum of 99% enriched acetic acid. Dashed lines in (a) and (b) indicate the integration windows.

8. ^{13}C Hyperpolarization quantification using external ^{13}C reference solution

To achieve accurate quantification of ^{13}C hyperpolarization, we employed an external ^{13}C reference solution alongside our 72 mm ID ^{13}C resonator. This method provides a more feasible and precise calibration for the quantification. For a 1 ml sample of 80mM EA (^{13}C natural abundance of 1.1%) which was hyperpolarized to 30% at 7T, the signal enhancement factor (*SE*) was calculated to be *SE* ≈ 50,000, and the measured signal-to-noise ratio (SNR) for the hyperpolarized sample (SNR_{HP}) was approximately 40 (Figure S8). To determine the feasibility of detecting the thermally polarized sample after the SAMBADENA experiment, we calculated the number of averages (N) required to achieve an SNR_{limit} of 5, which is deemed sufficient to detect a thermally polarized sample.

The relation used was:

$$N = \left(\frac{SE}{SNR_{\mathrm{HP}}} SNR_{\mathrm{limit}}\right)^2 = \left(\frac{50{,}000}{40} 5\right)^2 = 39{,}062{,}500$$

Assuming a repetition time (T_{R}) of approximately 1 minute, the total acquisition time required to achieve this SNR would be 651,042 hours, i.e. 27,127 days or ≈100 years. For a ^{13}C enriched sample (i.e., ethyl-[1-^{13}C]pyruvate-*d*$_6$, 99% ^{13}C), the acquisition time is reduced by 8,100-fold, i.e. to ≈80 hours.

Considering the stability of the magnet and potential errors from integrating low SNR signals, we opted for an external ^{13}C reference solution to ensure more precise quantification. The reference solution was carefully prepared to similar volume of the hyperpolarized sample, positioned in the same location within the reactor, and measured with a repetition time $(T_{\mathrm{R}}) > 5 \times T_1$ relaxation time. For accurate daily polarization calculations, we measure the reference solution at the start of each experimental day.

9. References

1. Morris, G. A. & Toohey, M. J. Removal of “acoustic ringing” from NMR spectra. *Journal of Magnetic Resonance (1969)* **63**, 629–633 (1985).
2. Ding, Y. *et al.* Rapidly Signal-enhanced Metabolites for Atomic Scale Monitoring of Living Cells with Magnetic Resonance. *Chemistry Methods* **2**, (2022).
3. Korchak, S., Yang, S., Mamone, S. & Glöggler, S. Pulsed Magnetic Resonance to Signal-Enhance Metabolites within Seconds by utilizing para-Hydrogen. *ChemistryOpen* **7**, 344–348 (2018).
4. Korchak, S., Emondts, M., Mamone, S., Blümich, B. & Glöggler, S. Production of highly concentrated and hyperpolarized metabolites within seconds in high and low magnetic fields. *Phys. Chem. Chem. Phys.* **21**, 22849–22856 (2019).
5. Pravdivtsev, A. N. *et al.* Parahydrogen-induced polarization and spin order transfer in ethyl pyruvate at high magnetic fields. *Sci Rep* **12**, 19361 (2022).
